# Causal risk factors for asthma in Mendelian randomization studies: A systematic review and meta‐analysis

**DOI:** 10.1002/clt2.12207

**Published:** 2022-11-07

**Authors:** Heidi Mikkelsen, Eskild Morten Landt, Marianne Benn, Børge Grønne Nordestgaard, Morten Dahl

**Affiliations:** ^1^ Department of Clinical Biochemistry Zealand University Hospital Køge Denmark; ^2^ Department of Clinical Medicine, Faculty of Health and Medical Sciences University of Copenhagen Copenhagen Denmark; ^3^ Department of Clinical Biochemistry Rigshospitalet Copenhagen University Hospital Copenhagen Denmark; ^4^ Department of Clinical Biochemistry Herlev and Gentofte Hospital Copenhagen University Hospital Herlev Denmark

**Keywords:** allergy, epidemiology, genetics, pulmonary function, risk factors

## Abstract

**Background:**

Several risk factors for asthma have been proposed; however, the causality of these associations is sometimes unclear. Mendelian randomization is a powerful epidemiological approach that can help elucidate the causality of risk factors. The aim of the present study was to identify causal risk factors for asthma through Mendelian Randomization studies.

**Methods:**

A systematic search of PubMed and EMBASE was conducted, to identify studies investigating risk factors for asthma or respiratory allergies through Mendelian Randomization. When two or more studies investigated the same risk factor a meta‐analysis was conducted. Of 239 studies initially identified, 41 were included.

**Results:**

A causal association between adiposity and adult asthma risk was found in 10 out of 12 studies with a summary risk ratio of 1.05 per kg/m^2^ increase in BMI (95% CI: 1.03–1.07). Puberty timing (*n* = 3), alcohol (*n* = 2), and linoleic acid (*n* = 1) had causal effects on asthma risk, while vitamins/minerals (*n* = 6) showed no consistent effect on asthma. The effect of smoking on adult asthma conflicted between studies. Several of the significant associations of asthma with immune related proteins (*n* = 5) and depression (*n* = 2) investigated through multiple traits analyses could generally benefit from replications in independent datasets.

**Conclusion:**

This systematic review and meta‐analysis found evidence for causal effects of adiposity, puberty timing, linoleic acid, alcohol, immune related proteins, and depression on risk of asthma.

## 1 INTRODUCTION

Several risk factors for asthma have been proposed, and the disease is increasingly recognized as a complex multifactorial disease with both multiple environmental and genetic risk factors. This heterogeneous disease is now more considered an umbrella disease gradually being classified into different phenotypes and endotypes based on clinical, demographic, trigger‐related and pathological factors.^1^, ^2^ Despite around 300 million people suffering from asthma worldwide, the mechanism and etiology of asthma are not yet fully understood. There is an urgent need for disentangling risk factor causation from correlation in the case of asthma and respiratory allergies in order to obtain better understanding of the mechanisms and etiology of asthma.^3^, ^4^, ^5^ This could allow targeted risk factor prevention for asthma and allergy with high impact on the asthma burden and the quality of life for patients with asthma.

Observational studies can be used to understand the extent and strength of association between risk factors and asthma, and to indicate novel associations; however, results from observational studies sometimes suffer from confounding and/or reverse causation. Randomized clinical trials can control for both confounding and reverse causation, but randomized trials can be unethical or infeasible to conduct ‐ exemplified by asking people to smoke or drink alcohol. In these cases, Mendelian randomization (MR) studies provide a well‐established design for investigating causal risk factors in diseases such as asthma independent of other risk factors (Supplementary Figure S1).^6^ The concept of MR builds on the random segregation of alleles from parents to offspring. These randomly allocated types of alleles are consequently not expected to be associated with any confounders except those on the causal pathway between genotypes and outcome. Also, as risk factors encountered later in life cannot influence the genetic makeup of an individual, MR also avoids the problem of reverse causation. MR thereby constitutes a naturally determined randomized controlled trial in which the two random allocated groups are divided on the basis of genetic variants comparable to treatment and placebo groups.

We conducted a systematic review and meta‐analysis of MR studies investigating any causal risk factor for asthma and other respiratory allergies to (1) provide an overview of the current knowledge of causal risk factors for asthma and respiratory allergies, and (2) to clarify where more research is needed.

## 2 MATERIALS AND METHODS

### 2.1 Search strategy

A systematic literature search was performed to identify studies using the MR approach to investigate possible risk factors for asthma and respiratory allergies. Studies were identified through electronic searches of PubMed (1966‐July 29th, 2021) and EMBASE (1947‐ July 29th, 2021) using broad search terms and MeSH terms such as “Mendelian randomization analysis”, “Asthma” and “Allergy”. For PubMed the following search string was used: ((Asthma) OR (Hay Fever) OR (Respiratory Hypersensitivity) OR (Allergy) OR (Atopy) OR (atopic) OR (Airway Hyperresponsiveness) OR (Rhinitis) OR (Eosinophilic Bronchitis)) AND (Mendelian randomization). The full search strategy is provided in the Supplementary Method S1, Search strategy. Reference lists within identified studies and reviews were screened to identify potentially missed studies from the initial search.

### 2.2 Study selection and data collection

Studies using MR to investigate the association of any potential risk factor for asthma or other respiratory allergies were included. The use of MR as a method had to be specified for inclusion of the studies identified. Thus, studies using genetic variations as proxies without using MR design were not included. Studies not directly related to asthma or respiratory allergy were excluded. We additionally excluded reviews, statistical, methodological and theoretical papers, editorials, commentaries, letters and conference abstracts. The search was not language restricted. For further details see supplementary methods S1.

### 2.3 Risk of bias/quality score assessments

The risk of bias of the MR studies was assessed through a quality assessment scheme built from references.^7^, ^8^, ^9^, ^10^ The quality assessment scheme takes both the MR limitations and the MR assumptions into account (Supplementary Table 1) and allows potentially biased studies to be evaluated on fair basis. The quality scores range from 0 to 13 points, with 13 points awarded to studies with high statistical power and low risk of bias. “High sample size” was defined based on a power of 0.8 or above in the study, a sample size comparable to similar studies with power of 0.8 or above, or an assessment of what sample size would approximately be needed to achieve a power of 0.8 or above. Biology of the instrument was included in the quality score in accordance with Burgess et al.,^9^ who recommended that variant selection for MR should be based on variants having biological relevance to the exposure. The quality score system was used to weight studies such that the results from studies of high quality, and those from meta‐analysis, were reported earlier and described more elaborately, than were results from single studies with low quality score.

The certainty of evidence for each potential asthma risk factor was assessed using the Grading of Recommendations, Assessment, Development and Evaluation (GRADE) framework.^11^ The MR studies were initially assigned high quality of evidence as in the paper by Kim et al.,^12^ the quality was downgraded if risk of bias, inconsistency, indirectness, imprecision or publication bias were causes of serious concerns (one level) or very serious concerns (two levels). Evaluation of risk of bias was adapted to the MR study design, whereas the remaining risks were assessed as recommended by Cochrane.^11^

### 2.4 Meta‐analysis

Meta‐analyses were performed on studies investigating risk factors for asthma using STATA version 12.0. When two or more studies investigating the same risk factor were identified, the data was pooled using the random‐effects model in the *metan* package. Heterogeneity was assessed through the I^2^ test.

## 3 RESULTS

**FIGURE 1:**
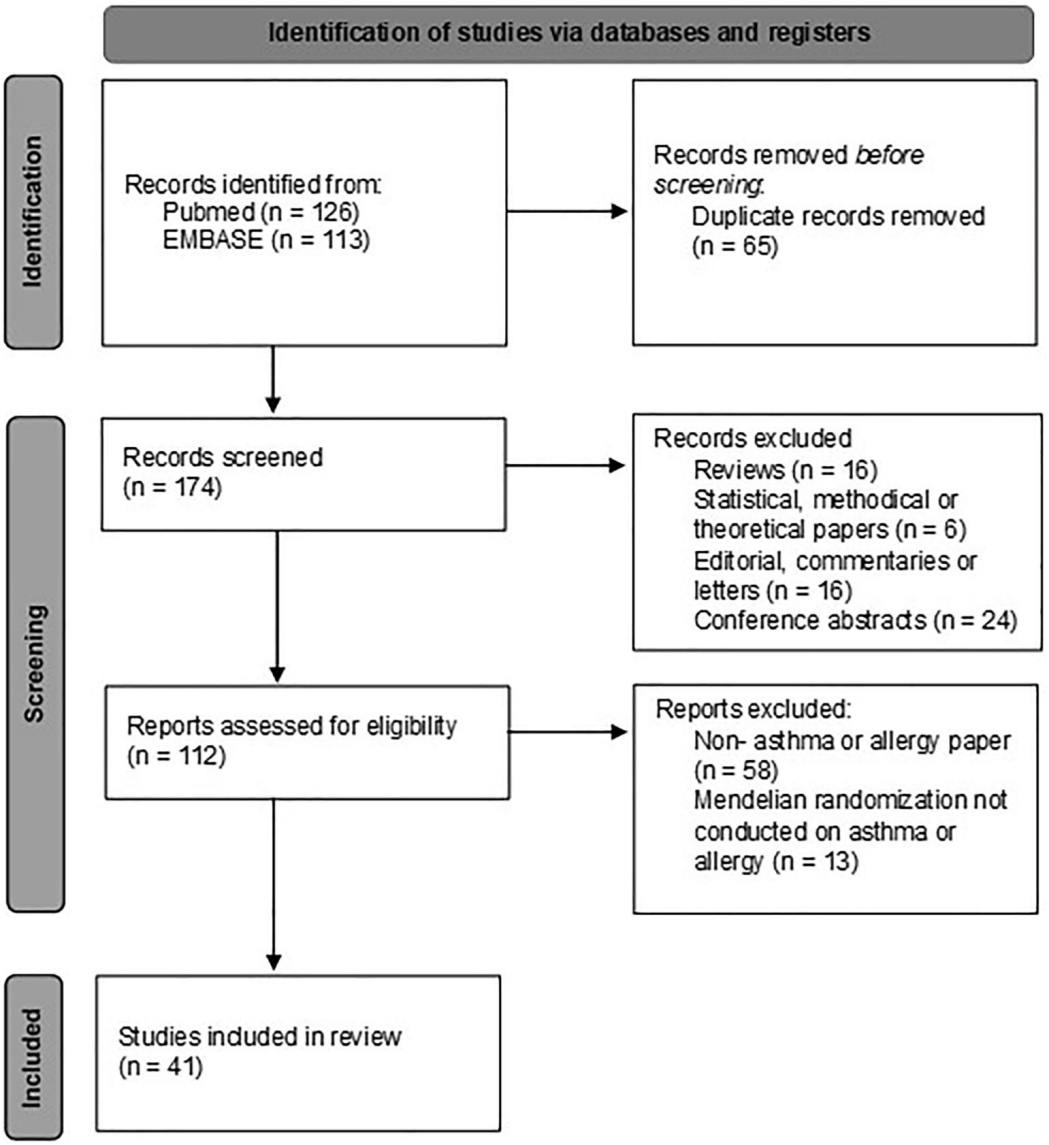
Preferred Reporting Items of Systematic Review and Meta‐analyses (PRISMA) flow diagram. 239 studies were initially identified in the electronic database search of PubMed and EMBASE and 41 studies were included

239 studies were initially identified in the electronic database search of PubMed and EMBASE. After removal of duplicates (65 studies), 174 studies were screened based on title and abstract. Of these, 112 were found to be eligible for full text screening and detailed evaluation, based on the inclusion and exclusion criteria. Forty one studies were included in the review. A summary of the search flow is shown in Figure 1.

### 3.1 Risk of bias assessment

The risk of bias score ranged from 3 to 13 points, with the lowest scores awarded to some of the oldest MR studies (Supplementary Table 2). The oldest studies were carried out before sensitivity analyses were developed and so are low quality for this reason. As a case in Granell et al.,^13^ the sample size was small with only 5364 participants and there were no information on MR method or sensitivity analyses. The highest scores were awarded to wellpowered and ‐designed studies such as the studies from Ha et al., Shen et al., and Au Yeung et al.^14^, ^15^, ^16^

### 3.2 Level of evidence

The level of evidence for each potential asthma risk factor identified from the MR studies was evaluated by the GRADE framework in Supplementary Table 3. About a quarter of the meta‐analysis/studies were supported by high evidence certainty. About a third were graded with moderate and another third were graded with low evidence certainty. A low grading was often given to studies which screened for multiple outcomes followed by MR, or studies with serious concerns for risk of bias.

### 3.3 Definition of asthma and subtypes

Most asthma definitions were based on self‐reported physician diagnosed asthma, some included wheezing and/or asthma treatment, others relied on hospital contact. Classification of atopic and non‐atopic asthma was based on different methods including fractional exhaled nitric oxide measurements,^17^ having allergic rhinitis in combination with use of allergy medication or reported allergic symptoms,^18^ positive skin prick test,^13^ or investigated allergic sensitization defined as specific IgE positivity to one or more inhalant allergens^19^ (Supplementary Results S1).

## 4 CAUSAL RISK FACTORS FOR ASTHMA AND RESPIRATORY ALLERGY

Forty one studies examined a potential causal association between risk factors and asthma or respiratory allergy through MR analysis. Most studies were based on European individuals with primarily European ancestry. Two studies used the Taiwan Children Health study and thereby an Asian population. Some of the studies used multiple cohorts to increase sample size. The oldest study was from 2007; however, most of the studies were relatively recent.

### 4.1 Anthropometry

**FIGURE 2:**
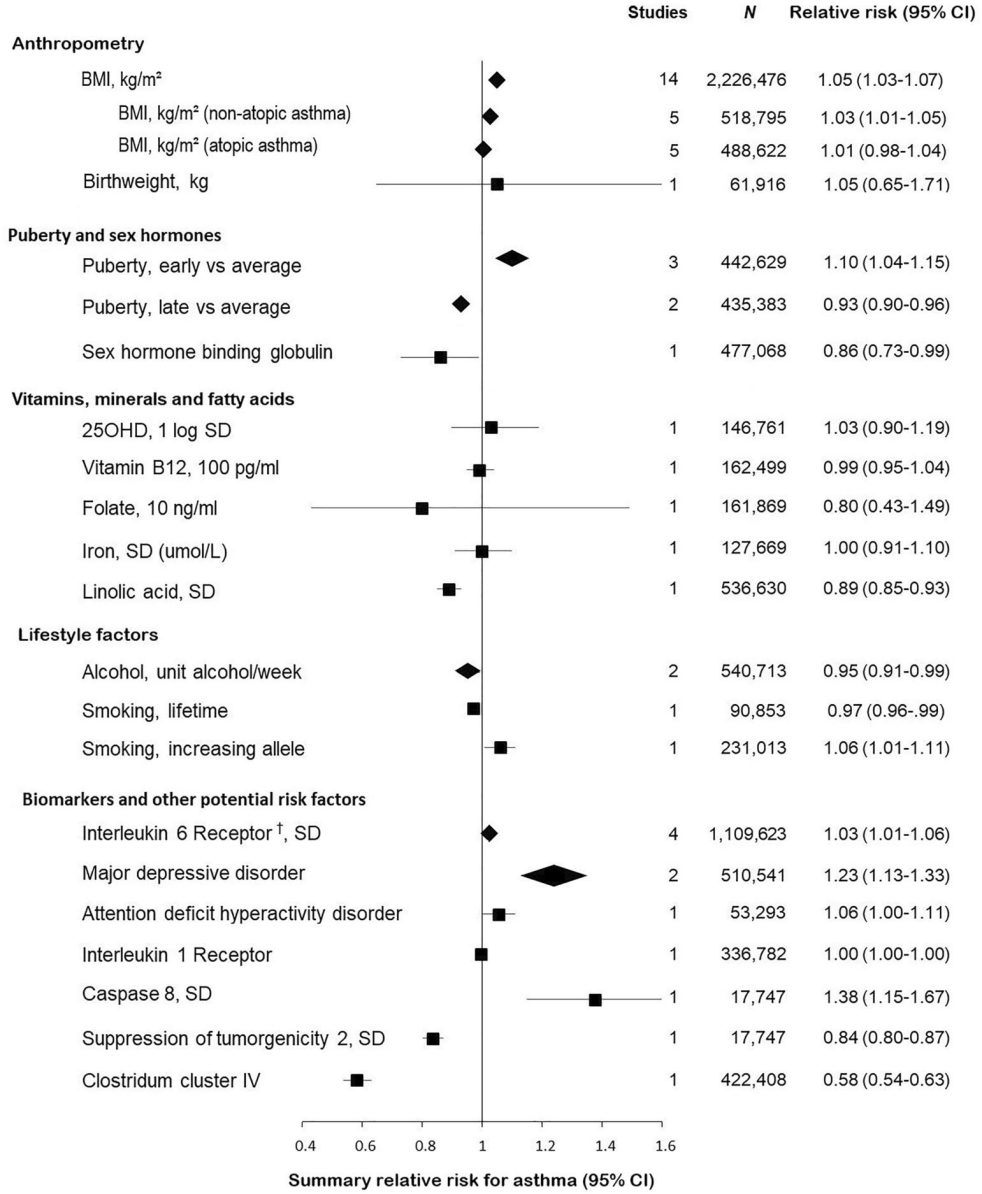
Overview of causal effects of risk factors on asthma risk in Mendelian randomization studies. Meta‐analysis was performed when two or more studies investigated the same risk factor on a comparable scale. Diamonds represent summary estimates with 95% confidence intervals. Squares represent individual studies with the 95% confidence interval represented by the line passing through the square. The solid vertical line represents the reference line of no risk (relative risk = 1). † Meta‐analysis of studies investigating different forms of the interleukin 6 receptor. Folkersen et al investigated the interleukin 6 receptor alpha subunit, while Rosa, McGowan, and Raita et al. based their MR studies on measurements of the soluble form of the interleukin 6 receptor. For information on the individual meta‐analysis please see Supplementary Figures 2–9

Twelve studies investigated the potential casual association between adiposity and asthma (Table 1). Eight studies were conducted in adults and three in children. Ten out of the 12 studies showed a causal association between genetically higher BMI and increased risk of asthma.^15^, ^16^, ^17^, ^18^, ^20^, ^21^, ^22^, ^23^, ^24^, ^25^ The pooled risk ratio (RR) for asthma for 1 kg/m^2^ increment in BMI was 1.05 (95% CI: 1.03–1.07) (Figure 2, Supplementary Figure 2), with overall evidence graded of high certainty (Supplementary Table 3). The pooled RR for non‐atopic asthma for 1 kg/m^2^ increment in BMI was 1.03 (1.01–1.05) compared to 1.01 (0.98–1.04) for atopic asthma (Figure 2, Supplementary Figures 3 and 4), with overall evidences graded of moderate certainty (Supplementary Table 3).

**TABLE 1:** Studies investigating for causal association between body mass index/birthweight and asthma using MR

First author, year	Method	Cases No.	Asthma risk measure	Atopic asthma and non‐atopic asthma	Quality score
Ha et al. (2021)^16^	Inverse‐variance weighted	35,926 asthma cases and 227,924 controls of European	OR for adult onset moderate‐severe asthma: 1.12 (95% CI: 1.07–1.16), adult‐onset mild OR: 1.06 (1.03–1.08), childhood onset mild OR: 1.02 (0.99–1.05), childhood onset moderate to severe OR: 1.10 (1.04–1.17)		13
Hyppönen et al. (2019)^25^	Two‐sample IVW	325,404 white British individuals (UKB)	OR for asthma per SD (4.1 kg/m2) higher BMI 1.32 (95% CI 1.17–1.48)		12
Xu et al. (2019)^24^	Two‐sample bi‐directional MR (Fixed effect meta‐analysis)	322,154 of European ancestry	OR for asthma per unit (SD) increase in BMI 1.18 (95% CI: 1.11–1.25), *p* = 2*10^−8^. In UKB, BMI mean = 27.43 and SD = 4.785		12
Skaaby et al. (2018)^20^	One‐sample IV (2SLS)	162,124 Europeans >16 years	OR for forever asthma per 1 kg/m^2^ increase in BMI: 1.07 (95% CI: 1.03–1.10). OR for hay fever per 1 kg/m^2^ increase in BMI 0.99 (95% CI: 0.96–1.01)		12
Zeng et al. (2019)^27^	Two‐sample IVW MR	EGG GWAS consortium for birth weight (143,677 European) and GERA cohort for asthma (61,916 European)	OR for adult asthma per unit SD change of offspring birth weight 1.02 (95% CI: 0.84‐1.24)		12
Au Yeung et al. (2021)^15^	Inverse‐variance weighted	401,837 British of European ancestry (UKB)	OR for asthma per SD increase in childhood BMI 1.10 (95%CI: 0.99–1.22) and for adult BMI (per SD) 1.33 (95% CI: 1.25–1.43)		11
Ҫolak et al. (2016)^55^		85,437 Danish individuals >20 years	OR for any asthma per increase in BMI unit (1 kg/m^2^) 1.08 (95% CI: 0.98‐1.19)		11
Sun et al. (2020)^18^	Wald method	56,105 Norwegians >20 years	OR for doctor‐diagnosed asthma per SD (4.1 kg/m^2^) increase in genetically determined BMI. Overall 1.49 (95% CI: 1.14–1.94), women 1.64 (1.17–2.30), men 1.31 (0.85–2.03)	OR per SD increase in genetically determined BMI forever asthma: 1.25 (0.89–1.77) and 1.42 (1.0–‐1.85)	11
Zhu et al. (2018)^22^	Generalized summary MR	162,030 of European ancestry (GERA and UKB)	OR for asthma risk per SD (3.98 kg/m^2^) associated with BMI: 1.35 (95% CI: 1.20–1.51), and associated with height 0.90 (95% CI: 0.87–0.93). OR for allergic rhinitis associated with height 0.96 (95% CI: 0.92–0.99)		10
Zhu et al. (2020)^21^	Summary data‐based MR	457,690 of European ancestry (GIANT and UKB)	OR for late‐onset asthma after 16 years per SD increase in BMI (*p*‐value): 1.21 (*p* = 6.3 × 10^−7^)	OR for atopic asthma per SD increase in BMI: 1.20 (*p* = 0.04) and for non‐atopic: 1.10 (*p* = 8.4 × 10^−6^)	9
Chen et al. (2019)^17^	2SLS regression	5138 Taiwan children age 10‐11	IV estimated RR for active asthma per unit z‐score for BMI overall: 1.04 (95% CI: 1.00–1.07). Males: 1.06 (95% CI: 1.01–1.12) and females: 1.02 (95% CI: 0.97–1.06)	1.02 (1.00–1.04) and 1.03 (1.00–1.06)	8
Granell et al. (2014)^23^	Two‐stage GMM	4835 UK children age 7 and 4298 children age 9	RR for current asthma associated with BMI at 7 years 1.55 (95% CI: 1.16–2.07) and 9 years 1.38 (95% CI: 1.06–1.80)	For 7 years 0.98 (0.92–1.05) and 1.08 (1.02–1.14). For 9 years 0.96 (0.91–1.03) and 1.05 (0.99–1.11)	8
Chen et al. (2021)^26^	2SLS method	6130 Taiwan children	OR for active asthma at age 17 per unite increase in z‐score for BMI at birth weight 1.00 (95% CI: 0.82‐1.16) and at age 17: 1.08 (0.96‐1.22)		7

Abbreviations: BMI, Body Mass Index; CI, Confidence interval; GERA, Genetic Epidemiology Research on adult health and aging; IV, Inverse variance; MR, Mendelian Randomization; OR, Odds ratio; RR, Relative Risk; SD, Standard deviation; 2SLS, Two stages Least Squares; Two‐stage GMM, Two‐stage Generalized moment method; UK, United Kingdom; UKB, United Kingdom Biobank.

Au Yeung et al. (quality score 11) showed that high childhood BMI (before age of 18) tended to increase risk of lifetime asthma, and also found a significant strong effect of adult high BMI on risk of asthma, proposing the effect of childhood high BMI on asthma to be mediated via a higher risk of having a high BMI in adulthood for children with a high BMI.^15^ Two MR studies (quality scores 8 and 8) found, respectively, a small tendency for a significant result showing that higher adiposity led to mid‐childhood (7–11 years) asthma, with a trend towards a stronger association for non‐atopic compared to atopic asthma.^17^, ^23^ Another study from Chen et al. (quality score 7) showed no causal effect of higher BMI from birth to 17 years on the risk of asthma at age 17.^26^

Zeng et al.^27^ (quality score 12, certainty of evidence graded high) investigated birth weight, as it is often used as a proxy for early life development and potentially an effect on adult diseases as well; however, Zeng et al. found no causal association between adult asthma and birth weight.

### 4.2 Puberty and sex hormones

Two studies investigated the association between timing of puberty and asthma^28^, ^29^ (Table 2). Both MR studies showed that early pubertal maturation was causally associated with higher risk of asthma.^28^, ^29^ The pooled RR for asthma for early puberty compared to normal puberty was 1.10 (1.04–1.15) (Figure 2, Supplementary Figure 5), evidence graded of moderate certainty (Supplementary Table 3). Additionally, Minelli et al. (quality score 12) found that late menarche/voice breaking was associated with a protective causal effect on asthma with ORs of 0.92 (0.89–0.97) in women and 0.93 (0.87–0.99) in men^29^ (Figure 2, Supplementary Figure 6).

**TABLE 2:** Studies investigating for causal association between puberty/sex hormones and asthma using MR

First author, year	Method	Cases No.	Active asthma	Quality score
Minelli et al. (2018)^29^		243,316 women and 192,067 men white aged 40‐69 years (UKB)	OR for asthma associated with early menarche versus normal menarche 1.08 (95% CI: 1.04–1.12) and late menarche versus normal menarche 0.93 (95% CI: 0.89–0.97).	12
			OR for asthma in men for early voice breaking 1.07 (95%CI: 1.00‐1.16) and for late voice breaking 0.93 (95%CI: 0.87‐0.99)	
Arathimos et al. (2019)^30^	Two‐sample MR (IVW)	62,285 asthma cases and 414,783 controls of European ancestry	OR for asthma associated with genetically increased SHBG 0.86 (95% CI: 0.74–1.00)	10
Chen et al. (2020)^28^	2SLS regression	7246 Taiwan children age 11,12 and 17 years	OR for active asthma associated with early pubertal maturation 1.18 (95% CI: 1.08–1.28)	8

Abbreviations: 2SLS, Two stages Least Squares; CI, Confidence interval; IVW, Inverse variance Weighted; OR, Odds ratio; SHBG, Sex Hormone Binding Globulin; UKB, United Kingdom Biobank.

Arathimos et al. (quality score 10, certainty of evidence graded moderate) suggested a protective effect of genetically increased sex hormone‐binding globulin (SHBG) on asthma (Table 2).^30^ As children with lower SHBG levels have been shown to start puberty earlier,^31^ this association fits well with the findings of Minelli et al. and Chen et al.^28^, ^29^

### 4.3 Vitamins, minerals and fatty acid levels

Seven MR studies investigated the effect of differing vitamin, mineral and fatty acid levels on asthma/allergy risk (Table 3, Figure 2). Two MR studies examined the association between vitamin D (25OHD) levels and, respectively, risk of asthma and respiratory allergies. Feng et al. (quality score 12) found no evidence for a causal association between serum 25OHD levels and allergic rhinitis or sensitization risk.^32^ In the case of asthma, no evidence was found for a casual role of low 25OHD on asthma or childhood onset asthma risk in the study by Manousaki et al.^33^ (quality score 7).

**TABLE 3:** Studies investigating for causal association of vitamins, minerals and fatty acids in asthma using MR

First author, year	Method	Cases No.	Asthma	Quality score
Vitamin D				
Feng et al. (2021)^32^	Two sample MR with random‐effects IVW method	29,859 allergic rhinitis cases and 267,670 controls of European‐ancestry. For allergic sensitization: 8040 cases and 16,441 controls	OR for risk of allergic rhinitis due to genetically decreased 25OHD 0.96 (95% CI: 0.78–1.18) and for allergic sensitization 1.06 (95% CI: 0.69–1.63)	12
Manousaki et al. (2017)^33^	Summary statistics of two‐sample MR	25,471 asthma cases and 121,290 controls + 7047 childhood asthma cases and 7961 controls all European	OR for risk of asthma per SD decrease in log‐transformed 25OHD: 1.03 (95% CI: 0.90–1.19), *p* = 0.63. OR for childhood onset asthma 0.95 (0.69–1.31), *p* = 0.76	7
Serum levels of B12 and folate				
Skaaby et al. (2018)^19^	Two‐sample MR (IVW)	162,499 of European ancestry	OR for asthma per 100 pg/ml vitamin B12 0.99 (95% CI: 0.95–1.04) and for hay fever 1.02 (0.98–1.05). OR for asthma per 10 ng/ml folate 0.80 (95% CI: 0.43–1.49) and for hay fever 0.74 (0.45–1.21)	10
Granell et al. (2008)^13^	Unknown MR method	5364 children(age 7) and 7356 mothers UK citizens	ORs for children risk of atopy with MTHFR TT (low folate levels) 0.92 (95% CI: 0.72–1.17) and for the risk of allergy in mothers 1.02 (0.88–1.2)	3
Iron				
Huang et al. (2019)^34^	Two‐sample MR (IVW)	19,954 asthma cases and 107,715 controls of European ancestry	OR for overall asthma per SD increase in iron (μmol/L) 1.00 (95% CI: 0.91–1.10). OR for childhood onset asthma was 1.14 (95% CI: 0.94–1.39) and for late onset asthma 0.92 (95% CI: 0.67–1.25)	10
Bédard et al. (2018)^35^		6002 white children. Age 7–9 years	OR for asthma per SD increase of genetic iron score in mothers during pregnancy without iron supplementation in late pregnancy was 1.09 (95% CI: 0.97–1.22), *p* = 0.15. OR for atopy per SD increase in iron score for atopy was 1.08 (95% CI: 0.97–1.19) and for hay fever 0.98 (95% CI: 0.89–1.08) adjusted for iron supplementation during pregnancy	11
Linoleic acid				
Zhao et al. (2019)^36^	Meta‐analysis two sample MR (IVW)	408,961 from UKB + 127,669 from TAGC all of European ancestry	OR for asthma per SD increased LA was 0.89 (95% CI: 0.85–0.93), *p* = 8.5*10^−7^ from meta‐analysis of 2 MR studies respectively on UKB and TAGC data.	11

Abbreviations: 25OHD, 25‐hydroxy‐vitamin D; CI, Confidence interval; IVW, Inverse variance Weighting l; LA, Linoleic Acid; MR, Mendelian Randomization; MTHFR, Methylenetetrahydrofolate reductase; OR, Odds ratio; SD, Standard deviation; UK, United Kingdom; UKB, United Kingdom biobank.

Skaaby et al. (quality score 10) investigated the association of serum levels of B12 and folate with asthma and hay fever risk, but found no evidence for causation.^19^ Neither did Granell et al. (quality score 3) when they investigated the association between *methylenetetrahydrofolate reductase* (MTHFR) C677 T, an important enzyme of folate metabolism (as indicator for low folate levels), and atopy and allergy risk in children and their mothers.^13^

Furthermore, the MR analysis of Huang et al. (quality score 10) did not find any evidence for an effect of iron status on asthma risk.^34^ This absence of association remained when investigating exclusively childhood onset and late onset asthma. The maternal iron status during pregnancy and its association with offspring asthma risk was examined by Bédard et al. (quality score 11): no causal effect was found on asthma, but weak evidence was demonstrated for an association between lower iron status during pregnancy and lower forced expiratory volume in the first second (FEV1) and forced vital capacity (FVC) in offspring.^35^

A MR study conducted by Zhao et al. (quality score 11) examined the role of linoleic acid in asthma and found an inverse association of genetically predicted linoleic acid with asthma.^36^ Thus, linoleic acid may have a protective role towards asthma (although certainty of evidence was graded low, supplementary Table 3).

### 4.4 Lifestyle factors

Six studies investigated the effect of lifestyle factors on the risk of asthma^14^, ^37^, ^38^, ^39^, ^40^, ^41^ (Table 4). Three of the studies investigated the causal effect of alcohol consumption using the rs1229984 gene variant as instrument, and allergic diseases and/or adult onset asthma as outcomes.^37^, ^38^, ^39^ The pooled RR per 1 unit/week higher alcohol intake showed a small protective role on the risk of adult asthma (Figure 2, Supplementary Figure 7) with certainty of evidence graded moderate (Supplementary Table 3). No evidence was found for a causal effect of high alcohol consumption on the risk of allergic diseases, although similar decreased risk estimates to those for asthma were seen.^36^, ^37^ Shaheen et al. (quality score 5) investigated the association of prenatal alcohol exposure with risk of childhood asthma, finding no causal relationship.^39^

**TABLE 4:** Studies investigating for causal association between lifestyle factors and asthma using MR

First author, year	Method	Cases No.	Asthma/allergy	Quality score
Shen et al. (2020)^14^	MR IVW	14,085 asthma participants and 76,768 controls of European ancestry	OR for asthma associated with increased lifetime smoking was 0.97 (95% CI 0.96–0.99), *p* = 1.77E‐04	11
Skaaby et al. (2017)^40^	MR fixed effect meta‐analysis IVW	231,020 of European ancestry	OR for asthma in current smokers associated to the smoking increasing allele was 1.06 (95% CI 1.01–1.11), *p* = 0.02. OR for hay fever per smoking increasing allele in current smokers was 0.958 (95% CI 0.920–0.998)	8
Skaaby et al. (2019)^38^	MR IVW	442,256 of European ancestry aged ≥16 years	OR per unit of alcohol/week consumed for asthma was 0.90 (95% CI 0.79–1.02). For hay fever 0.91 (95% CI: 0.81–1.02) and allergic sensitization 0.97 (95% CI: 0.80–1.17)	7
Lomholt et al. (2016)^37^	MR	98,457 white Danish subjects	OR for allergic disease including asthma for slow alcohol metabolizers was 0.93 (95% CI: 0.86–1.00). OR for allergic disease including asthma per one alcoholic drink/week 0.96 (95% CI: 0.92–1.00)	7
Bryan et al. (2021)^41^	Two MR	Hispanic and Latino adults with high rice consumption *n* = 2522 (*n* = 1127 ever smokers and *n* = 1395 never‐smokers)	No association between metabolism of arsenic and lifetime or current asthma. OR for past asthma associated with metabolism of arsenic for each percentage‐point increase in percent of inorganic arsenic: 1.40 (95% CI 1.05–1.86) and of percent of dimethylarsinate 0.87 (95% CI 0.77–0.98) in never smokers.	7
Shaheen et al. (2014)^39^	MR	4755 white mothers and children	OR for childhood onset asthma associated with maternal ADH1B genotype was 0.98 (95% CI 0.66–1.47) and for hay fever 1.11 (95% CI 0.71–1.72)	5

Abbreviations: ADH1B, Alcohol dehydrogenase 1B; CI, Confidence interval; IVW, Inverse Variance Weighting; MR, Mendelian Randomization; OR, Odds ratio; SD, Standard deviation.

Shen et al. (quality score 11, certainty of evidence graded high) found that increased lifetime smoking based on 124 genetic variants reduced risk of adult onset asthma.^14^ Skaaby et al.(quality score 8) examined the casual effect of smoking on adult asthma and hay fever, finding the investigated smoking‐increasing allele to be associated with lower hay fever risk and higher adult asthma risk in current smokers.^40^ With these conflicting results, the causal effect of smoking on asthma development remains inconclusive.

Bryan et al. (quality score 7) investigated the potential causal association between metabolism of arsenic, and asthma, in participants consuming high amounts of rice, finding no effect of arsenic on lifetime or current asthma risk but finding an effect in never smokers with past asthma diagnoses.^41^

### 4.5 Biomarkers and other potential risk factors

Different biomarkers have been investigated as causal risk factors for asthma and respiratory allergic diseases (Table 5). Four studies showed a positive association between the risk of asthma and the interleukin 6 receptor (IL‐6R).^42^, ^43^, ^44^, ^45^ Raita, Rosa, and McGowan et al. based their MR studies on measurements of the soluble form of IL‐6R,^43^, ^44^, ^45^ while Folkersen et al. investigated the IL‐6R alpha subunit.^42^ The meta‐analysis of the four studies resulted in a pooled RR for asthma with an increase in IL‐6R of 1.03 (1.01–1.04) (Figure 2, Supplementary Figure 8), certainty of evidence graded high (Supplementary Table 3).

**TABLE 5:** Studies investigating for causal association between biochemical factors and asthma using MR

First author, year	Method	Cases No.	Asthma/allergy	Quality score
Raita et al. (2021)^44^	IVW meta‐analysis method	394,256 subjects of European ancestry	OR per one SD increment in inverse‐rank normalized soluble IL‐6R level 1.02 (95% CI: 1.01–1.03)	12
Lyons et al. (2019)^50^	IVW	534 EPGA cases and 6688 controls	Finding a causal effect of eosinophil count on EGPA risk, *p* < 7.7 × 10^−12^	12
Groot et al. (2020)^53^	PheWAS and Wald estimates	UK biobank: 422,408 unrelated individuals	Wald beta effect estimate for atopy including asthma associated with genetically determined higher levels of *clostridium* cluster IV ‐0.54 (SE: 0.04), (RR: 0.58 (95% CI: 0.54–0.63)), *p* = 1.45*10^−37^	11
Amini et al. (2018)^51^	Two‐stage, least square (2SLS)	13,301 in Lifeline cohort, 967 asthma cases	No significant association for eosinophil count and asthma risk	10
Zhu et al. (2019)^47^	Generalized summary data‐based Mendelian randomization	UK biobank and Psychiatric genomics consortium: 347,481 European controls and 46,889 asthma cases	Beta effect estimate for MR for asthma associated with MDD: *β* = 0.21, (SE = 0.049), *p* = 1.80*10^−5^ and associated with ADHD: *β* = 0.054, (SE = 0.026), *p* = 0.036	9
Mulugeta et al. (2020)^46^	PheWAS and random effects IVW	UK biobank: 337,536 white British	OR for asthma associated with MDD 1.23 (95% CI: 1.06–1.44), and OR for painful respiration 1.28 (95% CI: 1.14–1.44)	9
Rosa et al. (2019)^45^	PheWAS MR and two sample MR IVW	180,129 asthma cases and 180,709 controls of European ancestry	OR for asthma in causal inference with sIL‐6R 1.03 (95% CI: 1.02–1.04), *p* = 5.62*10^−8^	9
Valette et al.^49^	IVW	56,167 asthma cases and 352,255 controls from UKB	Identified 50 blood expressed genes to be causally associated to risk of asthma including MHC, FADS1 and SMAD3	9
Huang et al. (2020)^48^	Two‐sample MR Weighted mode	Australian birth cohort (CAS) 234 individuals followed from birth to 10 years	In resting T cells, log odds decrease per SD increase in BTN3A2 for asthma = −0.056. For childhood asthma: −0.047. For adult‐onset asthma: −0.039. For allergic rhinitis: −0.044	9
McGowan et al. (2019)^43^	Wald ratio	UK biobank: 38,791 asthma cases and 297,991 controls	Beta effect estimate for asthma: sIL‐6R: 0.0103 (SE: 0.0027), *p* = 0.0001, IL‐1R: −0.0035 (SE: 0.0018), *p* = 0.0451	7
Folkersen et al. (2020)^42^	IV Wald ratio estimate	Total of up to 21,758 individuals primary European; average per‐protein sample size was 17,747	Beta for asthma per SD protein for *ci*s‐SNPs for CASP‐8: 0.32 (95%CI: 0.14–0.51), *p* = 6.1e−04, IL‐6RA: 0.07 (95% CI: 0.04–0.09), *p* = 6.3e−07, ST2: −0.18 (95% CI: −0.22 to −0.14), *p* = 4.0e−19	7
Arathimos et al. (2017)^52^	Wald ratio	ALSPAC and GABRIEL consortium	No significant evidence for causal effects of increased DNA methylation on asthma.	6

Abbreviations: ADHD, Attention deficit hyperactivity disorder; BTN3A2, Butyrophilin Subfamily 3 Member A2; CAS, Childhood Asthma Study; CASP‐8, Caspase eight; CI, Confidence interval; EGG, Early Growth Genetics; FADS1, Fatty acid desaturase one; GERA, Genetic Epidemiology Research on Aging; IL‐1R, Interleukin 1 receptor; IL‐6RA, Interleukin 6 receptor alpha; IVW, Inverse variance Weighting; MDD, Major Depressive Disorder; MHC, Major histocompatibility complex; MR, Mendelian Randomization; OR, Odds ratio; SD, Standard deviation; SE, Standard Error; sIL‐6R, Soluble Interleukin 6 receptor; SMAD3, SMAD family member 3; ST2, Suppression of tumorigenicity two; UKB, United Kingdom biobank.

Two MR studies investigated the association between major depressive disorder (MDD) and adult asthma among other disease outcomes.^46^, ^47^ The MR analyses were performed either through phenome wide association study (PheWAS) followed by MR or through generalized summary data MR. Both found a causal association between MDD and adult asthma risk, pooled RR 1.23 (1.13‐1.33) (Figure 2, Supplementary Figure 9). Mulugeta et al. (quality score 9) further found an association between MDD and painful respiration (OR: 1.28 (1.14‐1.44))^46^ and Zhu et al. (quality score 9) a small causal effect of ADHD on asthma risk.^47^

Huang et al. (quality score 9) used MR to investigate how various genetic variants associated with gene expression in neonatal immune cells affected risk of asthma and allergy.^48^ They found that increased expression in resting T cells of Butyrophilin Subfamily 3 Member A2 (BTN3A2) was causally associated with decreased risk of asthma and allergic rhinitis. BTN3A2 belongs to the butyrophilin family of proteins, which has diverse function in the immune system, including immune modulation, which helps in establishing self‐tolerance.

Valette et al. (quality score 9) investigated 431 blood expressed genes through MR and identified 50 blood expressed genes that were causally associated to asthma.^49^ Folkersen et al. (quality score 7) investigated potential drug targets through MR and found a causal protective role of ST2, a member of the interleukin 1 receptor (IL‐1R) family, against asthma and a positive association between CASP‐8 and the risk of asthma.^42^ McGowan et al. (quality score 7) showed a negative association between IL‐1R and asthma; however, the MR study was performed on a single genetic variant and had no sensitivity analysis to evaluate potential bias.^43^

Lyons et al. (quality score 12) showed a strong causal effect of eosinophil count on eosinophilic granulomatosis with polyangiitis (EGPA) risk. EGPA is characterized by a prodromal period with asthma.^50^ Amini et al. (quality score 10) investigated whether higher blood eosinophil count could cause asthma and found no such evidence.^51^ However, they also stated that their study indicated weak instrument bias and power limitations on the pulmonary outcomes, thus warranting further studies. Arathimos et al. (quality score 6) used a two‐sample bi‐directional MR to investigate the association between DNA methylation and asthma and found a causal effect of asthma on DNA methylation but no evidence that DNA methylation had an effect on asthma risk.^52^ Arathimos used levels of methylation at specific sites as exposure in their bi‐directional MR analyses.

Groot et al. (quality score 11) investigated the associations between human genetic determinants of the gut microbiome and health and disease through a PheWAS MR, finding an association between genetically determined higher levels of *clostridium* cluster IV (also called the *Clostridium* leptum group) and a lower risk for atopy including asthma (RR: 0.58 (0.54–0.63)); however, this association did not remain after sensitivity analyses.^53^

## 5 DISCUSSION

This systematic review and meta‐analysis included 41 MR studies, investigating any causal risk factors for asthma and other respiratory allergies. We found evidence for causal effects of adiposity, puberty timing, linoleic acid, alcohol, immune related proteins, and depression on risk of asthma (Figure 2); however, understanding of causal risk factors for asthma would benefit from more well conducted MR studies.

Most of the MR studies were published within the last couple of years emphasizing the increased use and acknowledgement of MR as a strong study design. Generally, the studies had low risk of bias with consideration of both population stratification, statistical power, Hardy‐Weinberg equilibrium, strength of the genetic instruments, and pleiotropy. The most recent studies were often awarded high scores in our quality assessment scheme accentuating an improvement in how MR studies are performed and published in recent years. The asthma definition was mostly based on self‐reported doctor diagnosed asthma, whereas the classification of atopic asthma and allergy were based on more diverse criteria across the studies, making comparisons more difficult. Studies based on PheWAS were generally rated lower by GRADE, as these studies would benefit from replication and had concerns for risk of bias often with no sensitivity analysis.

Strong evidence was found for a causal association between high BMI and adult asthma resulting in an increased summary risk ratio for asthma of 1.05 (95%CI, 1.03–1.07). The effect of a high adult BMI increasing asthma risk is supported by previous studies, including a meta‐analysis of prospective epidemiologic studies, finding that overweight and obesity are associated with incident asthma in a dose dependent manner.^54^ The pathophysiological mechanism from high BMI to increased risk of asthma is still unclear; however, a possible mechanism could be obesity‐related increased inflammation or immune response, but restrictive physiology due to mechanical factors is also plausible. In support of the latter, Çolak et al.^55^ suggested that it was wheezing rather than high BMI per se that was causally related to increased risk of asthma, and that wheezing in consequence may have resulted in (over)diagnosis of asthma in individuals with high BMI. Whether a high childhood BMI also has a causal effect on childhood onset asthma is still unclear. The 2 MR studies investigating childhood BMI deviated on number, ethnicity and age of the children investigated, which could influence the results. As the body undergoes large changes during mid‐childhood (6–11 years) and puberty, it is possible that the effect of high BMI on asthma risk also changes during this period. This might complicate comparison of studies across mid‐childhood. Au Yeung et al. suggested that the effect of high childhood BMI on asthma risk was mediated via a larger risk of being overweight in adulthood.^15^

A higher causal risk for asthma was found for early puberty and a protective role against asthma for late puberty in meta‐analyses (summary risk ratios: 1.10, 1.04–1.15 and 0.93, 0.90–0.96, respectively). Whether the asthma risk associated with the timing of puberty is due to the shift in sex hormones or other factors is unclear. However, a previous MR study found a beneficial effect of late menarche on lung function in adulthood,^56^ underlining that the timing of puberty have an effect of the lungs. Another MR study found a tendency for a lower risk of asthma with a higher SHBG concentration. One must bear in mind that the use of genetic variants as proxies for exposures in MR study designs, does not enable examination of critical periods in life and the causal effect of SHBG could arise from other periods of life than puberty. No casual associations were found for birth weight on risk of asthma.

Despite the importance of vitamins and minerals on the immune system^57^ and a widely discussed potential effect on asthma and respiratory allergy,^58^, ^59^, ^60^ no associations were reported between different vitamins/minerals and risk of asthma in the included MR studies. Few studies were carried out for each exposure and in many power is likely to be an issue so it is difficult to rule out these exposures. MR studies from Mao et al. and Hysinger et al., published respectively as a letter and a “to the editor study”, and thereby not included in this review, support the findings of the included MR studies regarding no causal effect of vitamin D levels on asthma risk.^61^, ^62^

Linoleic acid, on the other hand, might have a causal protective effect on asthma risk. A previous MR study also showed a protective role of linoleic acid towards other autoimmune diseases,^63^ suggesting a potential mechanism through the immune system. The included MR study also finds an association between genetically predicted linoleic acid and eosinophil and neutrophil counts in blood, supporting the causal effect of linoleic acid on asthma via leukocyte traits. However, Amini et al. found no causal effect of blood eosinophil count on risk of asthma.^51^

The meta‐analysis showed a protective effect of alcohol on adult asthma risk (summary risk ratio: 0.95, 0.91‐0.99). The 2 MR studies on alcohol, however, were deemed as having a relative high risk of bias, had a minimum or no assessment of pleiotropy, and few sensitivity analyses were performed. In this systematic review and meta‐analysis a causal effect of smoking on risk of adult asthma could not be established, as 2 MR studies showed conflicting results. Skaaby et al., showed that current smoking increased the risk of asthma but seem protective against hay fever.^40^ In contrast, Shen et al. found a protective effect of smoking on risk of asthma.^14^ Previous studies have shown that asthmatic smokers have more severe symptoms and their lung function declines faster,^64^, ^65^ but whether or not smoking can cause asthma in adults in MR studies is poorly investigated. Studies have shown that cigarette smoke can both suppress and activate the immune response.^64^ These diverse effects of cigarette smoke could potentially be different depending on asthma endotypes, which could be part of the explanation for the conflicting results in the MR studies of smoking on risk of asthma. Skaaby et al. only showed an effect of smoking on asthma and hay fever in current smokers, whereas the effect in former, ever and never smokers was non‐significant. Smoke exposure has long term damaging effects on the lungs,^66^ and one would have expected, that at least a tendency to an effect should be apparent in former or ever smokers in the study from Skaaby et al.

Four MR studies found a positive association between, respectively, IL6RA and sIL6R and asthma resulting in an increased summary risk ratio for asthma of 1.03 (1.01–1.06). These findings are supported by previous studies reporting upregulation of IL6 receptor levels in asthma patients compared to controls^67^ and a genome wide association study finding an association between a variant in the IL6R gene and increased risk of asthma.^68^ Whether the effect of IL6R on asthma arise from the modulatory role of IL6 on the immune response or a more direct effect of for example, sIL‐6R on CD4 T cells or airway epithelial cells is still unresolved.^69^

Two independent studies found an association between MDD and adult asthma risk resulting in an increased summary risk ratio of 1.23 (1.13‐1.33); however, both as part of a general screening and the results would therefore benefit from independent replication. In PheWAS studies a negative association between *clostridium* cluster IV and the risk of atopy was found, but this association did not withstand sensitivity analysis and must be re‐examined.

Different immune related proteins were found to have causal effects on asthma risk. Among these, a protective role of altered levels of circulating ST2 proteins for risk of asthma and allergic rhinitis was found.^42^ The ST2 is a product of the IL1RL1 gene, a known asthma locus.^70^, ^71^ The variants included in this MR study comprise both asthma risk reducing and increasing variants. The ST2‐IL33 complex promotes a pro‐inflammatory type 2 response when membrane bound.^71^ However, when the ST2 is in soluble form the effect on asthma and inflammation is unclear. Previous studies have shown that the levels of serum ST2 increase proportionally with the severity of asthma exacerbations and is generally higher in asthma patients, suggesting an effect on asthma risk.^72^ Folkersen et al.^42^ found an association between CASP‐8 and high asthma risk. Few other studies have investigated the effect of CASP‐8 on asthma. One intervention study in mice reported that CASP‐8 can mediate an IL1 signal, which promotes a type 2 response and thereby increases pulmonary inflammation and allergic asthma^73^; whether this effect is the same in humans remain unresolved.

In addition, a protective effect of BTN3A2 expressed in resting T‐cells on asthma and allergic rhinitis was found.^48^ The included MR studies investigating immune related proteins all use methods investigating multiple traits simultaneously through either PheWAS, quantitative trait loci, or multiple‐trait co‐localization. These methods can be used to investigate large numbers of exposure‐outcome combinations in a hypothesis‐free manner. However, significant findings often should be treated more provisionally and may benefit from replication in an independent dataset with a more pre‐defined hypothesis.

This review and meta‐analysis provides an overview of the current knowledge of possible causal risk factors for asthma and respiratory allergy obtained through MR studies. There is a risk that this review is suffering from publication bias due to less publication of studies not reporting a positive association. Studies finding no causal association can help disprove some theories obtained from biased observational studies and are therefore important. In general, the understanding of risk factors for asthma and respiratory allergy would benefit from more and in some cases larger well conducted MR studies. Also, a stringent division of asthma into endotypes or phenotypes might help unravel the complex multifactorial disease and the possible heterogeneous effect of risk factors.

## 6 CONCLUSION

This systematic review found casual effects of BMI, pubertal timing, linoleic acid, alcohol, MDD, immune related proteins ST2, IL1R, CASP‐8, IL6R and BTN3A2 on asthma risk. The review showed conflicting results regarding the causal effect of smoking on asthma and inconclusive results about the effect of *clostridium* cluster IV. Effects of investigated risk factors for respiratory allergy followed the trends from asthma risk. MR studies investigating risk factors for asthma and respiratory allergy would help further clarify the causes of asthma and respiratory allergy, and help extend the results from this review.

## AUTHOR CONTRIBUTIONS

Mikkelsen: Conceptualization; equal, Data curation; equal, Formal analysis; equal, Methodology; equal, Project administration; equal, Writing – original draft; lead, Writing – review & editing; equal,  Morten Landt: Conceptualization; equal, Data curation; equal, Formal analysis; equal, Methodology; equal, Supervision; equal, Writing – review & editing; equal,  Benn: Conceptualization; equal, Data curation; equal, Formal analysis; equal, Methodology; equal, Supervision; equal, Writing – review & editing; equal,  Gronne Nordestgaard: Conceptualization; equal, Methodology; equal, Supervision; equal, Writing – review & editing; equal,  Dahl: Conceptualization; equal, Data curation; equal, Formal analysis; equal, Funding acquisition; lead, Methodology; equal, Project administration; equal, Supervision; lead, Writing – review & editing; equal.

## CONFLICTS OF INTEREST

None of the authors have any competing interests to declare.

## Supporting information

Supplementary Material

## Data Availability

Data sharing not applicable to this article as no datasets were generated or analysed during the current study.
