# Supplementary material for: Causal risk factors for asthma in Mendelian randomization studies: A systematic review and meta‐analysis
Source: Clin Transl Allergy. 2022 Nov 7;12(11):e12207. doi: 10.1002/clt2.12207 (PMC9640961; doi:10.1002/clt2.12207)
Supplement: Supplementary file 1 — Supplementary Material [file CLT2-12-e12207-s001.docx]

**Supplementary material**

**Causal Risk Factors for Asthma in Mendelian Randomization Studies: A Systematic Review and meta-analysis**

Heidi Mikkelsen^1,4^, Eskild M. Landt^1,4^, Marianne Benn^2,4^, Børge G. Nordestgaard^3,4^, Morten Dahl^1,4^

^1^Department of Clinical Biochemistry, Zealand University Hospital, Køge, Denmark; ^2^Department of Clinical Biochemistry, Rigshospitalet, Copenhagen University Hospital, Copenhagen, Denmark; ^3^Department of Clinical Biochemistry, Herlev and Gentofte Hospital, Copenhagen University Hospital, Herlev, Denmark; ^4^Faculty of Health and Medical Sciences, University of Copenhagen, Copenhagen, Denmark.

Correspondence: Morten Dahl, Professor, MD, PhD, DMSc, Department of Clinical Biochemistry,

Zealand University Hospital, Lykkebækvej 1, DK-4600, Køge, Denmark. E-mail:

[modah@regionsjaelland.dk](mailto:modah@regionsjaelland.dk)

**Table of Contents**

[**SM Methods, Search strategy** 2](#_Toc103238548)

[**SM Table 1, Quality assessment scheme** 4](#_Toc103238549)

[**SM Table 2, Quality score from individuals studies** 6](#_Toc103238550)

[**SM Results, Definition of atopic asthma** 7](#_Toc103238551)

[**SM Table 3, Overview of Grading of Recommendations, Assessment, Development and Evaluation (GRADE) framework** 8](#_Toc103238552)

[**SM Figure 1, Schematic overview of the principle of the Mendelian Randomization Design** 1](#_Toc103238553)1

[**SM Figure 2, Meta-analysis, BMI and asthma risk** 12](#_Toc103238553)

[**SM Figure 3, Meta-analysis, BMI and non-atopic asthma risk** 13](#_Toc103238554)

[**SM Figure 4, Meta-analysis, BMI and atopic asthma risk** 14](#_Toc103238555)

[**SM Figure 5, Meta-analysis, Early puberty and asthma risk** 15](#_Toc103238556)

[**SM Figure 6, Meta-analysis, Late puberty and asthma risk** 16](#_Toc103238557)

[**SM Figure 7, Meta-analysis, Alcohol and asthma risk** 17](#_Toc103238558)

[**SM Figure 8, Meta-analysis, Interleukin 6 Receptor and asthma risk** 18](#_Toc103238559)

[**SM Figure 9, Meta-analysis, Major depressive disorder and asthma risk** 19](#_Toc103238560)

## **SM Methods, Search strategy**

Search strategy

Titles and abstracts were screened independently by two investigators (HM and EL) to identify studies eligible for full text screening. Eligible studies were MR studies investigating any potential risk factor for asthma or other respiratory allergies. Reviews, statistical, methodological and theoretical papers, editorials, commentaries, letters and conference abstracts were excluded. Disagreements were resolved by consensus and by consulting a third reviewer when needed (MD). One reviewer (HM) performed the full text reviewing process and extracted the following data: name of first author, year of publication, sample size, characteristic of population/cohort, MR design, exposure and outcome investigated and findings. Further, the various definitions of asthma and asthma subtypes were assessed, as this could have an impact on the interpretations of the results.

Search strategy in PubMed

((asthma) OR (Hay Fever) OR (Respiratory hypersensitivity) OR (allergy) OR (atopy) OR (atopic) OR (airway hyperresponsiveness) OR (Rhinitis) OR (eosinophilic bronchitis)) AND (mendelian randomization)

126 studies 29.07.2021 in PubMed

Search strategy in Embase

((asthma) OR (Hay Fever) OR (Respiratory hypersensitivity) OR (allergy) OR (atopy) OR (atopic) OR (airway hyperresponsiveness) OR (Rhinitis) OR (eosinophilic bronchitis)) AND (mendelian randomization)

113 studies in EMBASE 29.07.2021

1. Asthma*.mp
2. Hay fever*.mp
3. Respiratory hypersensitivity.mp
4. Allergy*.mp
5. Atopy*.mp
6. Atopic*.mp
7. Airway hyperresonsiveness.mp
8. Rhinitis*.mp
9. Eosinophilic bronchitis.mp
10. 1 or 2 or 3 or 4 or 5 or 6 or 7 or 8 or 9
11. Mendelian randomization.mp
12. Mendelian randomization analysis/
13. 11 or 12
14. 10 and 13

## **SM Table 1, Quality assessment scheme**

Quality assessment scheme

| **Score** | **Criteria** | |
| --- | --- | --- |
|  |  | |
|  | **Reporting of the MR method used to calculate the estimates** | |
| 1 | Clearly reporting of the MR method used to calculate the estimates | |
| 0 | No clearly mention of MR method used | |
|  |  | |
|  | **Population stratification** | |
|  | **One-sample MR** | **Two sample MR** |
| 1 | Ethnicity of study population considered, reported and explained | Alike ethnicity and populations characteristic reported and considered in the two groups |
| 0 | Unknown or mixed ethnicity of study population | Unknown or mixed ethnicity of one or both study populations |
|  |  | |
|  | **Sample size and Power** | |
| 1 | The study had high sample size and seemed well powered (power of 0.8 or above). | |
| 0 | Study was small – small sample size, maybe lack of power | |
|  |  | |
|  | **Ascertainment of diagnosis** | |
| 2 | Clearly describe objective criteria for diagnosis (outcome)  (*i.e.* *ICD-codes, measurement methods, questionnaire with doctor diagnosis)* | |
| 1 | Self-reported diagnosis of asthma or respiratory allergy | |
| 0 | Not described | |
|  |  | |
|  | **Hardy-Weinberg equilibrium** | |
| 1 | Hardy-Weinberg equilibrium checked for the SNPs, and if diverged, SNPs were excluded | |
| 0 | No check for Hardy-Weinberg equilibrium | |
|  |  | |
|  | **Assessments of genetic variant-exposure correlations** | |
| 2 | An assessment and consideration of genetic variant – exposure correlation and strength  (*i.e. F-statistic*, *partial r^2^, beta of linear regression)* | |
| 1 | Reported strength of association from literature with identical study population | |
| 0 | No reporting or discussion of genetic variant-exposure correlation/citation of association from article using a non-identical study population /no consideration of measured correlation | |
|  |  | |
|  | **Considerations and discussions of pleiotropy and its consequences for the results** | |
|  | **Biological effects of genetic variant explained and discussed** | |
| 1 | Potential biological effects of the genetic variant explained and discussed   - *Report from prior evidence i.e. summary statistic from GWAS meta analyses (*P*-value < 5 x 10^-8^)* - *Presenting biological support of the assumption* - *Canalization considered – requires comprehensive background knowledge* | |
| 0 | Biological effects not considered or reported | |
|  |  | |
|  | **Assessments of possible pleiotropy and linkage disequilibrium** | |
| 2 | Assessment and consideration of possible pleiotropy   - 1. *Use of “ positive” or “negative” controls*   2. *Investigation of the direction of bias in one sample MR i.e. Wald ratio*   3. *Winners curse (overestimating the effect of the SNP with lowest P-value as it often is the only SNP reported)*      1. *Wald method or ratio of coefficients*      2. *Two-sample MR analysis is a method to alleviate the issue of winners curse*   4. *Intercept test of MR-Egger*   5. *Literature search* | |
| 1 | Discussion of the plausibility of the used genotype in the particular study | |
| 0 | No assessment or consideration of pleiotropy | |
|  |  | |
|  | **Application of sensitivity analyses to evaluate consequences of pleiotropy** | |
| 2 | Multiple sensitivity analyses were performed   \| *Funnel plots* \| *Leave one out analysis* \| *Between-instruments heterogeneity Q test* \| *MR – Egger* \| \| --- \| --- \| --- \| --- \| \| *MR PRESSO* \| *Weighted median method* \| *Adjusted and non-adjusted regressions* \| *ZEMPA* \| \| *Sargan’s test* \| *Liberal and conservative analysis for polygenetic analysis* \| *Subgroup analysis* \| *Replication cohort or model validation* \| | |
| 1 | Few or one sensitivity analyses were performed | |
| 0 | Not used or described | |

## **SM Table 2, Quality score from individuals studies**

Overview of scores from the Quality assessment scheme

|  | **MR Methods reported** | | **Population stratification** | | **Sample size and power** | | **Ascertainment of diagnosis** | | | **Hardy-Weinberg equilibrium** | | **Genetic variant-exposure correlations** | | | **Biological effects of genetic variant** | | **Assessments of pleiotropy and LD** | | | **Sensitivity analyses to evaluate pleiotropy** | | | **Total** |
| --- | --- | --- | --- | --- | --- | --- | --- | --- | --- | --- | --- | --- | --- | --- | --- | --- | --- | --- | --- | --- | --- | --- | --- |
|  | 1 | 0 | 1 | 0 | 1 | 0 | 2 | 1 | 0 | 1 | 0 | 2 | 1 | 0 | 1 | 0 | 2 | 1 | 0 | 2 | 1 | 0 |  |
| Amini, 2018 (51) |  |  |  |  |  |  |  |  |  |  |  |  |  |  |  |  |  |  |  |  |  |  | 10 |
| Arathimos,2017 (52) |  |  |  |  |  |  |  |  |  |  |  |  |  |  |  |  |  |  |  |  |  |  | 6 |
| Arathimos, 2019(30) |  |  |  |  |  |  |  |  |  |  |  |  |  |  |  |  |  |  |  |  |  |  | 10 |
| AuYeung, 2020 (15) |  |  |  |  |  |  |  |  |  |  |  |  |  |  |  |  |  |  |  |  |  |  | 11 |
| Bédard, 2018 (35) |  |  |  |  |  |  |  |  |  |  |  |  |  |  |  |  |  |  |  |  |  |  | 11 |
| Bryan, 2021 (41) |  |  |  |  |  |  |  |  |  |  |  |  |  |  |  |  |  |  |  |  |  |  | 7 |
| Chen,2019 (17) |  |  |  |  |  |  |  |  |  |  |  |  |  |  |  |  |  |  |  |  |  |  | 8 |
| Chen,2020 (28) |  |  |  |  |  |  |  |  |  |  |  |  |  |  |  |  |  |  |  |  |  |  | 8 |
| Chen,2021 (26) |  |  |  |  |  |  |  |  |  |  |  |  |  |  |  |  |  |  |  |  |  |  | 7 |
| Çolak, 2016 (55) |  |  |  |  |  |  |  |  |  |  |  |  |  |  |  |  |  |  |  |  |  |  | 11 |
| Feng, 2020 (32) |  |  |  |  |  |  |  |  |  |  |  |  |  |  |  |  |  |  |  |  |  |  | 12 |
| Folkersen, 2020 (42) |  |  |  |  |  |  |  |  |  |  |  |  |  |  |  |  |  |  |  |  |  |  | 7 |
| Granell,2014 (23) |  |  |  |  |  |  |  |  |  |  |  |  |  |  |  |  |  |  |  |  |  |  | 8 |
| Granell, 2008 (13) | |  |  |  |  |  |  |  |  |  |  |  |  |  |  |  |  |  |  |  |  |  | 3 |
| Groot 2020 (53) |  |  |  |  |  |  |  |  |  |  |  |  |  |  |  |  |  |  |  |  |  |  | 11 |
| Ha, 2021 (16) |  |  |  |  |  |  |  |  |  |  |  |  |  |  |  |  |  |  |  |  |  |  | 13 |
| Huang, 2019 (34) |  |  |  |  |  |  |  |  |  |  |  |  |  |  |  |  |  |  |  |  |  |  | 10 |
| Huang, 2020 (48) |  |  |  |  |  |  |  |  |  |  |  |  |  |  |  |  |  |  |  |  |  |  | 9 |
| Hyppönen, 2019 (25) |  |  |  |  |  |  |  |  |  |  |  |  |  |  |  |  |  |  |  |  |  |  | 12 |
| Lomholt, 2016 (37) | |  |  |  |  |  |  |  |  |  |  |  |  |  |  |  |  |  |  |  |  |  | 7 |
| Lyons, 2019 (50) |  |  |  |  |  |  |  |  |  |  |  |  |  |  |  |  |  |  |  |  |  |  | 12 |
| Manousaki, 2017 (33) | |  |  |  |  |  |  |  |  |  |  |  |  |  |  |  |  |  |  |  |  |  | 7 |
| McGowan, 2019 (43) |  |  |  |  |  |  |  |  |  |  |  |  |  |  |  |  |  |  |  |  |  |  | 7 |
| Minelli, 2018 (29) |  |  |  |  |  |  |  |  |  |  |  |  |  |  |  |  |  |  |  |  |  |  | 12 |
| Mulugeta, 2019 (46) |  |  |  |  |  |  |  |  |  |  |  |  |  |  |  |  |  |  |  |  |  |  | 9 |
| Raita, 2021 (44) |  |  |  |  |  |  |  |  |  |  |  |  |  |  |  |  |  |  |  |  |  |  | 12 |
| Rosa, 2019 (45) |  |  |  |  |  |  |  |  |  |  |  |  |  |  |  |  |  |  |  |  |  |  | 9 |
| Shaheen, 2014 (39) |  |  |  |  |  |  |  |  |  |  |  |  |  |  |  |  |  |  |  |  |  |  | 5 |
| Shen, 2020 (14) |  |  |  |  |  |  |  |  |  |  |  |  |  |  |  |  |  |  |  |  |  |  | 11 |
| Skaaby, 2018 (20) |  |  |  |  |  |  |  |  |  |  |  |  |  |  |  |  |  |  |  |  |  |  | 12 |
| Skaaby, 2019 (38) |  |  |  |  |  |  |  |  |  |  |  |  |  |  |  |  |  |  |  |  |  |  | 7 |
| Skaaby, 2018(19) |  |  |  |  |  |  |  |  |  |  |  |  |  |  |  |  |  |  |  |  |  |  | 10 |
| Skaaby,2017 (40) |  |  |  |  |  |  |  |  |  |  |  |  |  |  |  |  |  |  |  |  |  |  | 8 |
| Sun, 2020 (18) |  |  |  |  |  |  |  |  |  |  |  |  |  |  |  |  |  |  |  |  |  |  | 11 |
| Valette, 2021 (49) |  |  |  |  |  |  |  |  |  |  |  |  |  |  |  |  |  |  |  |  |  |  | 9 |
| Xu, 2019 (24) |  |  |  |  |  |  |  |  |  |  |  |  |  |  |  |  |  |  |  |  |  |  | 12 |
| Zeng,2020 (27) |  |  |  |  |  |  |  |  |  |  |  |  |  |  |  |  |  |  |  |  |  |  | 12 |
| Zhao, 2019 (36) |  |  |  |  |  |  |  |  |  |  |  |  |  |  |  |  |  |  |  |  |  |  | 11 |
| Zhu, 2020 (21) |  |  |  |  |  |  |  |  |  |  |  |  |  |  |  |  |  |  |  |  |  |  | 9 |
| Zhu, 2019 (47) |  |  |  |  |  |  |  |  |  |  |  |  |  |  |  |  |  |  |  |  |  |  | 9 |
| Zhu, 2018 (22) |  |  |  |  |  |  |  |  |  |  |  |  |  |  |  |  |  |  |  |  |  |  | 10 |

## **SM Results, Definition of atopic asthma**

Classification of atopic and non-atopic asthma was based on different methods throughout the MR studies:
Chen et al. used fractional exhaled nitric oxide measurements(17). Sun et al. defined atopic status as having allergic rhinitis in combination with use of allergy medication or reported allergic symptoms to pollen or pets(18). Zhu et al. defined atopic asthma as doctor diagnosed asthma with hay fever/allergic rhinitis or eczema(21), whereas Granell et al. determined the atopic status through a positive skin prick test. Skaaby et al. investigated allergic sensitization defined as specific IgE positivity to one or more inhalant allergens and investigated asthma and hay fever as individual traits(19), whereas Lomholt et al. referred to allergic disease, which was applied if participants responded yes on the question: “Does food, medicine, grass, flowers, animal hair or other things trigger asthma, rhinitis or eczema?”(37).

## **SM Table 3, Overview of Grading of Recommendations, Assessment, Development and Evaluation (GRADE) framework**

| **Risk factor for asthma *** | **Quality of the evidence** | **Notes** |
| --- | --- | --- |
| BMI | High | Variation of asthma diagnosis and age of populations but no serious concerns |
| BMI >< atopic asthma | Moderate | Various populations, small sample sizes |
| BMI >< non atopic asthma | Moderate | Low sample size in several studies, various populations |
| Birthweight | High | Small concerns for imprecision with only one study, wide confidence interval |
| Early puberty | Moderate | Concerns with inconsistency with I^2^ = 44,3 % and indirectness with different measurements of puberty timing |
| Late puberty | Moderate | Concerns with indirectness as two studies were conducted on different sex |
| Sex hormones | Moderate | Concerns with risk of bias, no sensitivity analysis, low sample size and no population stratification, furthermore a wide confidence interval. |
| Vitamin D | High | Findings do not exclude an association between asthma and the active form of vitamin D |
| Vitamin D levels on allergy risk | High | No serious concern |
| B12 | High | One study |
| Folate | Moderate | Wide confidence interval, one study |
| Low folates levels in mothers | Very low | Very serious concerns of risk of bias, wide confidence interval |
| Iron | High | One study |
| Maternal iron | Moderate | Only an association in the absence of iron supplementation in late pregnancy with a wide confidence interval based on one study |
| Linoleic acid | Low | The effect of endogenous LA could differ from the effect of dietary LA and genetic predictors only make a small difference to LA |
| Alcohol | Moderate | Low heterogeneity and few sensitivity analyses which raise some concerns for the risk of bias |
| Prenatal alcohol | Low | Few sensitivity analyses, small sample size and wide confidence interval |
| Lifetime smoking | High | Lifetime smoking is difficult to comprehend, results based on one study |
| Smoking increasing allele | Moderate | Based on one SNP, few sensitivity analyses, adverse events of smoking limit clinical significance |
| Arsenic | Moderate | Concerns for risk of bias, low sample size, few sensitivity analyses, no assessment of pleiotropy. Broad Confidence interval |
| IL6R | High | Various forms of IL6R |
| BTN3A2 | Low | Concerns for risk of bias and screening study |
| Major depressive disorder | Low | Low heterogeneity, MR study based on PheWas study |
| ADHD | Low | Lack of sensitivity analyses – risk of bias. MR study based on PheWas study |
| IL1R | Low | Lack of sensitivity analyses – risk of bias. MR study based on PheWas study |
| CASP8 | Very low | Small sample size, no ascertainment of diagnosis, no assessment of pleiotropy. MR study based on PheWas study. Wide confidence interval |
| ST2 | Low | Small sample size, no ascertainment of diagnosis, no assessment of pleiotropy. MR study based on PheWas study |
| Clostridum cluster IV | Low | Screening study risk of bias and indirectness |
| Eosinophil count on EGPA risk | Moderate | Low sample size, would benefit for a replication in a new dataset |
| Eosinophil count on asthma risk | Low | Low sample size, concerns of weak instrument bias, power limitations, concerns with proportion of eosinophil variance explained |

Certainty of evidence assessed using the Grading of Recommendations, Assessment, Development and Evaluation (GRADE) framework. The MR studies were initially assigned high quality of the evidence. Downgraded if risk of bias, inconsistency, indirectness, imprecision or publication bias was a cause of serious concerns (one level) or very serious concerns (two levels). *for risk of asthma unless other is stated. LA: Linoleic acid.

**SM Figure 1, Schematic overview of the principle of the Mendelian Randomization design.**


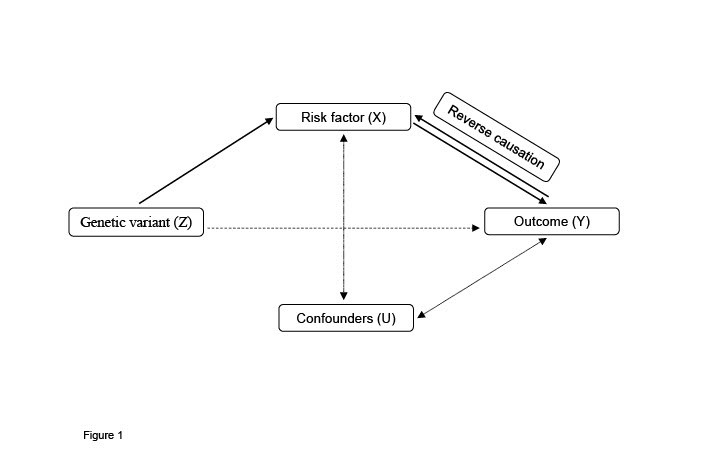


Mendelian Randomization uses exposure–associated genetic variants (Z) as instrumental variables to investigate the association with an outcome of interest (Y) in this case asthma or respiratory allergy. For the MR study to be valid, the only association between the genetic variant (Z) and the outcome (Y) must be via the risk factor (X). Three assumptions must be satisfied: The genetic variant (Z) is associated with the risk factor (X), the genetic variant is independent of confounders (U) and independent from other factors which may affect the outcome (Y).

## **SM Figure 2, Meta-analysis, BMI and asthma risk**

Meta-analysis of Mendelian randomized studies investigating the effect of higher BMI on the risk of asthma.

**Asthma**

Relative risk of asthma for one unit higher BMI (kg/m^2^). Chen et al., 2021 and Granell et al., 2014 include two risk estimates as the studies investigate two different age groups. The vertical red broken line and the rhombus represent the summary estimate of the overall effect. The lateral tips of the rhombus represent the 95 % confidence interval.

## **SM Figure 3, Meta-analysis, BMI and non-atopic asthma risk**

Meta-analysis of Mendelian randomized studies investigating the effect of higher BMI on the risk of non-atopic asthma.

**Non-atopic asthma**

Relative risk of non-atopic asthma for one unit higher BMI (kg/m^2^). Granell et al., 2014 include two risk estimates as the studies investigate two different age groups. The vertical red broken line and the rhombus represent the summary estimate of the overall effect. The lateral tips of the rhombus represent the 95 % confidence interval.

## **SM Figure 4, Meta-analysis, BMI and atopic asthma risk**

Meta-analysis of Mendelian randomized studies investigating the effect of higher BMI on the risk of atopic asthma.

**Atopic asthma**

Relative risk of atopic asthma for one unit higher BMI (kg/m^2^). Granell et al., 2014 include two risk estimates as the studies investigate two different age groups. The vertical red broken line and the rhombus represent the summary estimate of the overall effect. The lateral tips of the rhombus represent the 95 % confidence interval.

## **SM Figure 5, Meta-analysis, Early puberty and asthma risk**

Meta-analysis of Mendelian randomized studies investigating the causal association between early puberty and the risk of asthma.

Relative risk of asthma for early puberty compared to average puberty. Minelli et al., 2018 are represented two times as the first result are from MR analysis in girls (early menarch) and the second result are from MR analysis in boys (early voice breaking). The vertical red broken line and the rhombus represent the summary estimate of the overall effect. The lateral tips of the rhombus represent the 95 % confidence interval.

## **SM Figure 6, Meta-analysis, Late puberty and asthma risk**

Meta-analysis of Mendelian randomized studies investigating the causal association between late puberty and the risk of asthma.

Relative risk of asthma for late puberty compared to average puberty. Minelli et al., 2018 are represented two times as the first result are from MR analysis in girls (late menarch) and the second result are from MR analysis in boys (late voice breaking). The vertical red broken line and the rhombus represent the summary estimate of the overall effect. The lateral tips of the rhombus represent the 95 % confidence interval.

## **SM Figure 7, Meta-analysis, Alcohol and asthma risk**

Meta-analysis of Mendelian randomized studies investigating the causal association between alcohol and the risk of asthma.

Relative risk of adult asthma per one unit higher alcohol intake per week. The vertical red broken line and the rhombus represent the summary estimate of the overall effect. The lateral tips of the rhombus represent the 95 % confidence interval.

## **SM Figure 8, Meta-analysis, Interleukin 6 Receptor and asthma risk**

Meta-analysis of Mendelian randomized studies investigating the causal association between the interleukin 6 receptor and the risk of asthma.

Relative risk of asthma for increase in interleukin 6 receptor. Four studies are included whereas Folkersen et al investigated the association between the interleukin 6 receptor alpha subunit and risk of asthma, while Rosa, McGowan, and Raita et al. based their MR studies on measurements of the soluble form of the interleukin 6 receptor. The vertical red broken line and the rhombus represent the summary estimate of the overall effect. The lateral tips of the rhombus represent the 95 % confidence interval.

## **SM Figure 9, Meta-analysis, Major depressive disorder and asthma risk**

Meta-analysis of Mendelian randomized studies investigating the causal association between the major depressive disorder and the risk of asthma.

Relative risk of adult asthma for major depressive disorder compared to no depression. The vertical red broken line and the rhombus represent the summary estimate of the overall effect. The lateral tips of the rhombus represent the 95 % confidence interval.
